# The relationship between out of hours discharge and hospital outcome

**DOI:** 10.1186/2197-425X-3-S1-A360

**Published:** 2015-10-01

**Authors:** G Warnock, A Mackay, P Stenhouse

**Affiliations:** Intensive Care Department, Victoria Infirmary/NHS Greater Glasgow & Clyde, Glasgow, United Kingdom

## Introduction

Evidence suggests night-time discharges from the intensive care unit (ICU) are associated with poorer outcomes, and patients are at increased risk of clinical deterioration [1, 2]. Out-of-hours (OOH) discharges are used as a quality indicator for this reason. The Faculty for Intensive Care Medicine (FICM) and the Scottish Intensive Care Society (SICS) currently use different time periods to categorise a discharge as OOH [3, 4].

## Objectives

To assess if a difference exists in hospital outcome for patients discharged OOH from the ICU, using both SICS and FICM criteria, compared with daytime discharge.

## Methods

A WardWatcher^TM^search for unit discharges after 01/01/2014 (survivors) was performed. Two separate datasets based on FICM and SICS criteria were gathered. Data was excluded where there was missing APACHE-II, or hospital outcome data.

## Results

There were a total of 59 OOH discharges (SICS) vs. 27 (FICM). There was no significant difference between the mean age (58.6 vs. 56.7), APACHE score (20.2 vs. 20.1), or APACHE mortality prediction (37.1% vs. 38.3%) in SICS vs. FICM data sets. Tables one and two show a comparison of observed patients 'lived' vs. 'died' from both datasets, separated into daytime and OOH discharges.

The actual mortality rate for the FICM dataset was 11.4% (daytime) vs. 14.8% (OOH), compared with the SICS dataset 11.2% (daytime) vs. 13.6% (OOH). The datasets were analysed utilising a 2x2 contingency table and Fisher exact probability test with two tailed p-value as shown in tables one and two. The p-values were 0.75 (FICM) vs. 0.81 (SICS) showing there was no significant difference. There was a discrepancy of 2 patients between the FICM and SICS groups which is likely due to the search criteria employed.

## Conclusions

Our study showed that when looking at patients who have survived intensive care, the time of discharge does not significantly affect hospital survival regardless of whether we use the FICM or SICS criteria. One of the current quality indicators employed is an OOH discharge rate as it is supposed to correlate with mortality, however this does not appear to be the case. The SICS may soon be adopting the discharge criteria used by FICM, which in our study would decrease the number of patients classified as OOH discharges, but not produce a significant change in overall mortality.

**Table 1:** FICM dataset - observed patients lived vs. Died

FICM Dataset	In-hours (0700-2159)	OOH (2200-0659)
Lived	164	23
Died	21	4
Fisher exact probability test with two tailed p-value = 0.75

**Table 2:** SICS dataset - observed patients lived vs. Died

SICS Dataset	In-hours (0800-2000)	OOH (2001-0759)
Lived	134	51
Died	17	8
Fisher exact probability test with two tailed p-value = 0.81		
